# Telehealth to reduce compound pressures on the National Health Service and social care: a multi-methods study protocol

**DOI:** 10.1093/oodh/oqag032

**Published:** 2026-09-18

**Authors:** Jennifer Downing, Rosie Kaur, Saddaf Naaz Akhtar, Elizabeth M Camacho, Konstantinos Daras, Anna Cooper, Thais Caprioli, Yuxuan Yang, Brendan Collins, Peter Almond, Wesam Baker, Hilary Garret, Patricia Jamal, Benjamin Barr, Anthony G Marson, Eduard Shantsila

**Affiliations:** Department of Primary Care and Mental Health, Institute of Population Health, University of Liverpool, Waterhouse Building, Block B, Brownlow Street, Liverpool L69 3GF, United Kingdom; Mersey Care NHS Foundation Trust, Liverpool, Merseyside L34 1PJ, United Kingdom; Department of Public Health, Policy & Systems, Institute of Population Health, University of Liverpool, Waterhouse Building, Block F, Brownlow Street, Liverpool L69 3GF, United Kingdom; Department of Public Health, Policy & Systems, Institute of Population Health, University of Liverpool, Waterhouse Building, Block F, Brownlow Street, Liverpool L69 3GF, United Kingdom; Department of Public Health, Policy & Systems, Institute of Population Health, University of Liverpool, Waterhouse Building, Block F, Brownlow Street, Liverpool L69 3GF, United Kingdom; Mersey Care NHS Foundation Trust, Liverpool, Merseyside L34 1PJ, United Kingdom; Department of Primary Care and Mental Health, Institute of Population Health, University of Liverpool, Waterhouse Building, Block B, Brownlow Street, Liverpool L69 3GF, United Kingdom; Department of Public Health, Policy & Systems, Institute of Population Health, University of Liverpool, Waterhouse Building, Block F, Brownlow Street, Liverpool L69 3GF, United Kingdom; Department of Public Health, Policy & Systems, Institute of Population Health, University of Liverpool, Wheelan Building, Brownlow Street, Liverpool L69 3GF, United Kingdom; Mersey Care NHS Foundation Trust, Liverpool, Merseyside L34 1PJ, United Kingdom; Mersey Care NHS Foundation Trust, Liverpool, Merseyside L34 1PJ, United Kingdom; Faculty of Health & Medicine, Lancaster University, Bailrigg, Lancaster, Lancashire LA1 4YW, United Kingdom; National Institute for Health and Care Research Applied Research Collaboration North West Coast (NIHR ARC NWC), Waterhouse Building, Brownlow Street, Liverpool, Merseyside L69 3GL, United Kingdom; Department of Public Health, Policy & Systems, Institute of Population Health, University of Liverpool, Waterhouse Building, Block F, Brownlow Street, Liverpool L69 3GF, United Kingdom; Institute of Systems, Molecular and Integrative Biology, University of Liverpool, Crown Street, Liverpool, Merseyside L69 7ZB, United Kingdom; Department of Primary Care and Mental Health, Institute of Population Health, University of Liverpool, Waterhouse Building, Block B, Brownlow Street, Liverpool L69 3GF, United Kingdom

**Keywords:** telehealth service, long-term conditions, quality of life, health economics evaluation, unplanned healthcare utilisation, longitudinal design

## Abstract

Telehealth and remote monitoring are increasingly used in the management of long-term health conditions and can improve access to care. The Mersey Care Telehealth Service (MCTS) provides technology-enabled support for people with heart failure, diabetes, and chronic obstructive pulmonary disease. The service has been operating for over a decade; however, the COVID-19 pandemic has led to its rapid expansion. This study aims to gather evidence on the impact of the MCTS, explore barriers and enablers to effective service delivery and evaluate its cost-effectiveness. This study comprises of four work packages (WP). WP1: A retrospective observational analysis to evaluate whether the MCTS is associated with reduced unplanned healthcare utilisation and to examine how outcomes vary by sociodemographic characteristics and service delivery type; WP2: Longitudinal analyses to compare patient-reported outcomes, including mental health, quality of life, and wellbeing, between telehealth users and a comparator group of non-users who meet the eligibility criteria for telehealth; WP3: Remote semi-structured interviews with MCTS staff and patients will be held to explore the barriers and enablers influencing the effective service delivery; WP4: An economic evaluation will determine whether the MCTS is cost-effective compared with usual care, and will assess how cost-effectiveness varies by patient characteristics and service delivery type. This multi-method evaluation will provide robust and comprehensive evidence of the impact, equitable delivery, implementation approach, and cost-effectiveness of the MCTS. Insights seek to inform service improvement, commissioning decisions, and future telehealth strategy within the NHS.

## Introduction

Digital health technologies now form an essential pillar of modern healthcare delivery, rather than merely auxiliary tools. Within the United Kingdom (UK), remote monitoring and digital-first strategies are at the forefront of National Health Service (NHS) transformation thanks to national policy drivers like the NHS Long Term Plan (Department of Health and Social Care 2025, NHS 2019), Data Saves Lives (Department of Health & Social Care, 2022), and A Plan for Digital Health and Social Care (NHS England, 2019a; Department of Health & Social Care, 2022). Recent government and NHS policies focus on virtual care, remote monitoring (NHS England, 2025a), digital-first primary care (NHS England, 2019a), interoperability (NHS England, 2025b), and making it easier for people to get health information (Department of Health & Social Care, 2022). The recent NHS 10-Year Plan focusses on moving towards more preventative, digitally enabled services that are closer to home (Department of Health and Social Care, 2025). Digital communication and tools support synchronous (where patient data are reviewed remotely), asynchronous (where clinicians contact patients directly when assessment is required), and mixed-modality (integrating both asynchronous monitoring and synchronous clinical support) approaches to healthcare provision. The descriptions are often used interchangeably; however, they are conceptually different (Van Dyk, 2014). Telehealth refers to the use of digital and communication technologies to support health-related assessment, monitoring, education, self-management, and clinical decision-making at a distance, i.e., at the patients’ home (Van Dyk, 2014). Telemedicine typically describes synchronous consultations provided remotely rather than in person (Choudhury et al., 2022; Yadav et al., 2022); telemonitoring relates to the provision of home monitoring services, to collect health measurements, and/or structured remote (e.g., telephone) support (Scholte et al., 2023); Telecare refers to services typically provided to older adults with social care needs and can include monitoring of both health and behavioural factors (e.g., eating/drinking or falls) (Lassar and Hertelendy, 2024). Virtual clinics are pre-booked synchronous appointments that either replace or supplement out-patient care (Vashishth et al., 2026). Virtual wards deliver short-term, intensive, hospital-standard care to patients remotely in their own homes (Alhakem et al., 2025).

Telehealth is an important part of proactive management of health conditions and making care more accessible, which helps the NHS reach its goals of prevention, efficiency, and fair access. By enabling remote monitoring and care, it can help reduce pressure on NHS and social care services, improve multidisciplinary working, and enhance patient safety, particularly for remote or vulnerable populations. Although early uptake was variable, telehealth adoption increased substantially following the COVID-19 pandemic, aligning with the NHS England’s strategy to expand technology-enabled care and transform healthcare delivery (Department of Health & Social Care, 2022; NHS England, 2023).

### Evidence for telehealth in chronic disease management

Telehealth interventions are implemented across different stages of the chronic disease pathway, serving distinct purposes. The evidence base for digitally enabled care in long-term conditions is heterogeneous, partly because interventions are implemented at different stages of the patient pathway. Some interventions support early identification and risk stratification, while others focus on medication optimisation, routine follow-up, self-management support, early deterioration detection, or long-term disease management. In diabetes, for example, digital interventions support blood glucose monitoring, medication titration, lifestyle coaching, structured education, complication screening, and routine review. In chronic obstructive pulmonary disease (COPD) and heart failure (HF), remote monitoring focuses more directly on the early detection of deterioration, support for self-management, reduction of avoidable face-to-face contact, and timely escalation of care before crisis presentation. The Mersey Care Telehealth Service (MCTS) most closely aligns with clinician-led management of long-term conditions and the early detection of deterioration. In addition, it supports patient education and care coordination, rather than operating solely as a screening, appointment-based, or educational digital intervention.

There is evidence to support telehealth care for the management of HF (Pandor et al., 2013), COPD (McLean et al., 2011), and diabetes (Mabeza et al., 2022). For example, a systematic review identified five studies evaluating the impact of synchronous primary care telemedicine (i.e., real-time remote consultations) compared with in-person visits on diabetes outcomes. (Mabeza et al., 2022). Three studies included control groups following usual care with in-person visits. They showed that telemedicine led to significantly greater glycohemoglobin (HbA1c) reduction over 6 months, with similar or better HbA1c outcomes at 12 months. The other two studies showed improved HbA1c levels with telehealth but did not include comparisons with usual care. A UK randomised controlled study (Basudev et al., 2016) showed equivalent glycaemic control between the virtual clinic and standard care groups. However, the study was limited to six general practices in South London, which lacked established integrated telehealth service. The 2011 Cochrane systematic review of telehealth care for COPD found that telehealth care was associated with lower odds of emergency attendance over 12 months (OR 0.27, 95% CI 0.11–0.66, 3 trials, *n* = 449), and lower odds of hospital admission over 12 months (OR 0.46, 95% CI 0.33–0.65, 6 trials, *n* = 604) (McLean et al., 2011). There was no significant difference in 1-year mortality. Telehealth care was related to a better quality of life recorded by the St George’s Respiratory Questionnaire (two trials, *n* = 253, mean difference − 6.57, 95% CI –13.62–0.48 with minimum clinically significant difference being a change of −4.0). However, health economic evaluations remain scarce, and robust post-COVID-19 evidence is still lacking.

Evidence on whether telehealth care can reduce demand for urgent and emergency services is inconsistent (Janjua et al., 2021; McLean et al., 2010). A recent systematic review of remote monitoring in COPD included 29 studies (5,654 participants), but the studies were at high risk of bias, the certainty of evidence ranged from moderate to very low (Janjua et al., 2021). Early pre-pandemic evaluation of the Mersey Care (MC) model in the Liverpool area of Cheshire and Merseyside showed a small reduction in the use of emergency care services (the average treatment effect was 0.08 [95% CI, 0.05–0.11], corresponding to an average percentage decrease of 22.7%) (Van Berkel et al., 2019). Often health outcomes are unaffected by an intervention, findings show services producing small benefits for increased costs (Salisbury et al., 2017). Previous telehealth evaluations have often been limited by small pilot samples, short intervention periods, and insufficiently established services. Many studies also lack methodological rigour, including appropriate control groups and cost-effectiveness analyses. Importantly, few have examined differential effects across socioeconomic or patient groups, limiting evidence on telehealth’s potential to reduce health inequalities (Salisbury et al., 2017).

### The impact of COVID-19 on telehealth adoption

Although a substantial body of literature has examined the rapid expansion of telehealth during and following the COVID-19 pandemic, important evidence gaps remain. Existing studies have frequently focused on single clinical conditions, individual technologies, or narrowly defined outcomes. In contrast, relatively few studies have evaluated large-scale, routine telehealth services operating across multiple long-term conditions while simultaneously examining healthcare utilisation, patient-reported outcomes, health inequalities, implementation processes, and economic outcomes (Gajarawala and Pelkowski, 2021; Hanlon et al., 2017; Moynihan et al., 2021). Additionally, concerns regarding digital exclusion and unequal access to telehealth persist, particularly among socioeconomically disadvantaged and digitally underserved populations (Shaw et al., 2021; Williams and Shang, 2024). The need for robust evaluations of integrated telehealth programmes has therefore been highlighted by both NHS policy and digital health evidence frameworks (NHS, 2019; National Institute for Health and Care Excellence, 2022; NHS England, 2019b).

Healthcare services are experiencing unprecedented pressures, especially services in areas of high deprivation such as MC NHS Foundation Trust. Since 2011, MC has successfully implemented telehealth services for patients with long-term physical health conditions, particularly during the pandemic and amid increasing fiscal pressures on the NHS. Now, the Trust is considering extending telehealth to mental health services in line with NHS priorities (NHS England, 2023; 2024a). This study seeks to address important evidence gaps regarding the impact and cost-effectiveness of telehealth, particularly for marginalised and underserved populations. The initiative aims to improve patient outcomes, reduce health inequalities, and support healthcare professionals in managing workload demands, while generating critical evidence for the NHS on the overall impact of telehealth, service utilisation, and equitable access to care. Through this research, we aim to develop a comprehensive understanding of telehealth delivery and outcomes that can inform future service development, adaptation, and scale-up. Accordingly, the study is organised into four work packages (WPs):

**WP1:** To evaluate whether the MCTS is associated with reduced unplanned healthcare utilisation and to examine how outcomes vary by sociodemographic characteristics and service delivery type.

**WP2:** To assess patient-reported outcomes among MCTS users and examine how changes in quality of life, mental health, satisfaction, and wellbeing vary according to sociodemographic characteristics and service delivery models.

**WP3:** To identify and explore the barriers and enablers influencing the effective delivery of the MCTS.

**WP4:** To determine whether the MCTS is cost-effective compared with usual care, and to assess how cost-effectiveness varies by patient characteristics and service delivery type.

### Mersey care telehealth service

The MCTS is a complex, clinician-led remote monitoring intervention designed for patients with long-term conditions and/or an elevated risk of deterioration. The service was initially established in Liverpool in 2011 and delivered through an internally managed Mersey Care platform. In November 2017, following a tripartite partnership between Liverpool Clinical Commissioning Group (CCG), Mersey Care, and Docobo, the service transitioned to the Docobo remote monitoring platform. The service was subsequently expanded to additional localities, including St Helens (September 2021), Sefton (July 2022), Wirral (May 2023), and Cheshire West (February 2024). Across all localities, eligible patients include those with COPD, HF, and diabetes, as well as individuals with any combination of two of these conditions.

The service includes digital or telephone-supported submission of physiological observations and symptom data, asynchronous review by the telehealth team, patient education, self-management support, telephone contact, and escalation to primary, community, specialist, or urgent care services where clinically indicated. Patients may be monitored at varying levels of intensity depending on their condition, risk profile, clinical needs, and care pathway, with monitoring arrangements reviewed regularly by clinical staff. The intervention should therefore be understood as a whole-service model rather than the isolated effect of a digital platform. Consequently, any observed impact on hospital admissions, outpatient activity, or other healthcare utilisation outcomes may reflect the combined effects of technology-enabled monitoring, clinician-led case management, escalation processes, and supported self-management.

Using the Combined Intelligence for Population Health Action (CIPHA) dataset, a regional population health data platform that links routinely collected health and care records (including primary care, hospital, and community data) to support population risk stratification and service planning, patients eligible for the service are identified through established case-finding processes. Patients must be aged 18 years or older, have a confirmed diagnosis, and reside within a commissioned area. Eligibility and case-finding evolved during the study period. Prior to January 2024, identification of potentially eligible patients relied on the Welsh Predictive Model alongside existing clinical caseloads, referral pathways, and clinical judgement. From January 2024, the CIPHA Enhanced Case Finding Tool incorporating the Johns Hopkins Adjusted Clinical Groups (ACG) risk stratification system was introduced. Patients with an estimated six-month risk of emergency hospital admission of 37% or greater may be identified as suitable for telehealth review, subject to clinical assessment and pathway-specific eligibility criteria. As eligibility criteria and case-finding approaches changed over time, the clinical risk profile of patients enrolled in the service may have varied across the study period.

Although the Johns Hopkins ACG risk score (Johns Hopkins University, 2014) is used operationally to support case finding, it is not available for the wider eligible population included in the evaluation dataset and, therefore, cannot be used directly for comparator selection or matching. Consequently, analyses will adjust for available demographic, clinical, socioeconomic, locality, and prior healthcare utilisation characteristics as proxies for baseline risk.

Patients enrolled in MCTS receive a home remote-monitoring package (Doc@Home, Graphnet Ltd), with setup support provided in person, by telephone, or through written guidance. Patients submit daily symptom and physiological readings (e.g., oxygen saturation for COPD and weight and blood pressure for HF) via a mobile app or an NHS-provided device. Monitoring usually happens Monday through Friday for up to 5-months which represents the standard initial monitoring and review period within the telehealth pathway rather than a fixed maximum duration for all patients. Patients may be discharged, extended, stepped down, or re-enrolled according to clinical need and pathway-specific criteria.

MCTS is a complex, clinician-led intervention and variation may occur in monitoring intensity, alert frequency, escalation activity, and staff contact. A team of registered nurses (Band 6–8a) (NHS England, 2024b) and healthcare assistants monitor patient data daily. Alerts are generated using individualised, National Institute for Health and Care Excellence (NICE)-aligned thresholds based on physiological readings, symptoms, or missing data. All alerts are reviewed by the telehealth team and may prompt telephone assessment, self-management advice, referral, or escalation to urgent or emergency care, ensuring a clinician-led rather than fully automated response. Senior clinicians can also set individual thresholds when it makes sense to do so. Healthcare assistants check on patients who do not send in their readings and triage patient calls. Administrative staff support non-clinical activities such as enrolling patients onto the system (Mersey Care NHS Foundation Trust, 2025a; NHS England, 2025c). Patients receive written and video educational resources at enrolment. Patient-reported outcomes, including quality of life, medication adherence, mental health, self-management, and service use, are collected through online questionnaires. Communication is primarily via the app, with telephone contact used when required. Formal fidelity monitoring was not routinely collected; therefore, fidelity will not be formally evaluated. Where available, service activity measures will be reported descriptively to provide context on intervention delivery.

The MCTS was implemented incrementally across localities, conditions and platform configurations. This phased rollout is important for interpreting both service reach and the construction of comparator groups. A map illustrating the phased geographical rollout of MCTS is provided in Table 1 which summarises key changes in geography, eligibility, case finding, commissioned capacity, and platform use over the study period.

**Table 1:** Timeline of MCTS rollout, eligibility criteria and platform transitions.

Metric	Liverpool	Sefton	St Helens	Wirral	Cheshire West
Monitoring established date^*^	November 2017	July 2022	September 2021	May 2023	February 2024
Eligible conditions	COPD, HF, Diabetes (and any dual combination of these)	COPD, HF, Diabetes (and any dual combination of these)	COPD, HF, Diabetes (and any dual combination of these)	COPD, HF, Diabetes (and any dual combination of these)	COPD, HF, Diabetes (and any dual combination of these)
Monitoring intensities	Full clinical monitoring Light touch monitoring	Full clinical monitoring Light touch monitoring	Full clinical monitoring Light touch monitoring	Full clinical monitoring Light touch monitoring	Full clinical monitoring Light touch monitoring
Referral method (currently)^**^	CIPHA population tool (60%) GP referrals (10%) Step down/light touch (30%)	CIPHA population tool (18%) GP referrals (61%) Step down/light touch (21%)	CIPHA population tool (50%) GP referrals (0%) Step down/light touch (50%)	CIPHA population tool (81%) GP referrals (3%) Step down/light touch (17%)	CIPHA population tool (85%) GP referrals (8%) Step down (8%)
Case-finding approach	QOF conditions = COPD, HF Expanded diagnostic cluster = Type 2 diabetes w/o complication OR Type 2 diabetes w/ complication Age 18+ Nursing home care flag = ‘N’	QOF conditions = COPD, HF Expanded diagnostic cluster = Type 2 diabetes w/o complication OR Type 2 diabetes w/ complication Age 18+ Nursing home care flag = ‘N’	QOF conditions = COPD, HF Expanded diagnostic cluster = Type 2 diabetes w/o complication OR Type 2 diabetes w/ complication Age 18+ Nursing home care flag = ‘N’	QOF conditions = COPD, HF Expanded diagnostic cluster = Type 2 diabetes w/o complication OR Type 2 diabetes w/ complication Age 18+ Nursing home care flag = ‘N’	QOF conditions = COPD, HF Expanded diagnostic cluster = Type 2 diabetes w/o complication OR Type 2 diabetes w/ complication Age 18+ Nursing home care flag = ‘N’
Risk stratification method	Probability of Emergency Admission = 0.37	Probability of Emergency Admission = 0.37	Probability of Emergency Admission = 0.37	Probability of Emergency Admission = 0.37	Probability of Emergency Admission = 0.37
Commissioned capacity	2000	50	100	100	100
Platform used	Docobo (November 2017*—*April 2026) Luscii (April 2026—current)	Docobo (January 2018*—*April 2026) Luscii (April 2026—current)	Docobo (January 2018—April 2026) Luscii (April 2026—current)	Docobo (November 2017—April 2026) Luscii (April 2026—current)	Docobo (January 2018—April 2026) Luscii (April 2026—current)

**Abbreviations:** COPD—Chronic obstructive pulmonary disease; HF—Heart Failure; CIPHA—Combined Intelligence for Population Health Action; QOF—Quality and Outcomes Framework; GP—General Practitioner.

^*^These dates should be interpreted as the time period documented in project documentation for the pathway. However, the true time period where patients enrolled onto the service may be different.

^**^This is based on current patient referral routes as of the 13 July 2026.

## Methods

**Figure 1:**
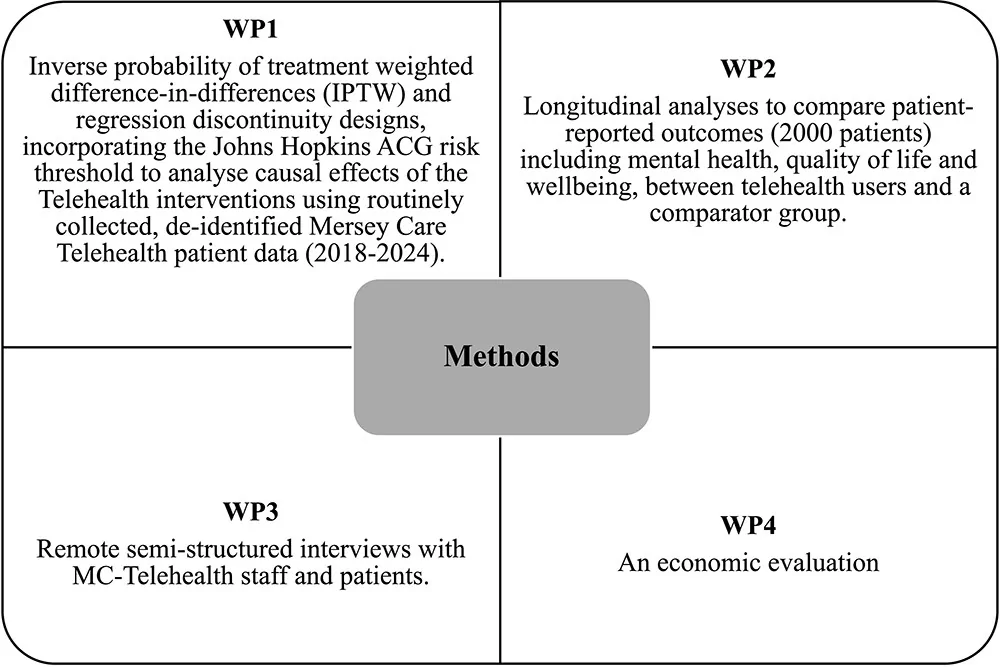
Methodological framework for the Mersey Care Telehealth Service evaluation study.

The methods section of the MCTS evaluation study is organised into four work packages (WPs), which are illustrated in Fig. 1.

**Work package 1:**  *To understand whether the Mersey Care Telehealth Service reduces the use of unplanned health care utilisation and whether there is a differential effect by sociodemographic group and service delivery type*

### Methods

Our primary outcome will be unplanned emergency hospital admissions (outcome 1), secondary outcomes will include: Accident & Emergency (A&E) attendances (outcome 2), 30-day readmission rates (outcome 3), admission to nursing/residential care home (outcome 4), and mortality (outcome 5). The study will analyse the seasonal impacts on hospital admissions during winter.

The study will use quasi-experimental methods and apply two complementary causal approaches: (i) inverse probability of treatment weighted difference-in-differences (IPTW-DiD) (Bettega et al., 2024; Chesnaye et al., 2022; Wei et al., 2023), this is our primary approach to robustly estimate causal effects of telehealth interventions using routinely collected data de-identified by Mersey Care between 2018 and 2024; (ii) To strengthen causal identification, we will also implement regression discontinuity (RD) designs (Thistlethwaite and Campbell, 1960), which are widely adopted in health research (Calonico et al., 2024; Thapa et al., 2025), based on eligibility thresholds derived from the Johns Hopkins ACG risk score (NHS England, 2025d), this is our secondary confirmatory approach. This aspect of the analysis is subject to the availability of Johns Hopkins data. MCTS is commissioned to provide daily care to up to 2,000 patients in Liverpool, 50 patients in Sefton, 100 patients in St Helens, 100 patients in Wirral, and 100 patients in Chesire, respectively. Since the service has not been available over the period to all groups in all parts of Cheshire and Merseyside to construct an appropriate comparator group who did not receive the telehealth service but had similar characteristics (confounders and pre-intervention outcomes) to the patients that did receive it (Barr et al., 2022).

The primary exposure is active enrolment onto the MCTS. Referral alone will not constitute exposure, as not all referred patients commence telehealth monitoring. For enrolled patients, the index date will be defined as the date on which active telehealth monitoring begins. To account for temporal variation in healthcare utilisation and service delivery, telehealth enrolment dates will be grouped into calendar quarters (Q1: January–March; Q2: April–June; Q3: July–September; Q4: October–December). Comparator patients will be selected from quarterly eligibility snapshots comprising individuals who were eligible for MCTS, alive, and registered during the same quarter but who did not enrol onto the service. Calendar quarter will be used as an exact matching variable within the propensity score matching process, ensuring that telehealth participants are compared only with eligible non-enrolled patients observed during the same time period. Additional demographic, clinical, risk, and baseline healthcare utilisation variables will be incorporated into the matching procedure. This approach aims to minimise temporal bias arising from changes in healthcare utilisation, seasonal pressures, COVID-19 recovery effects, admission practices, and service delivery over the study period. The primary analysis will therefore estimate the effect of active enrolment onto MCTS compared with eligible non-enrolment during the same calendar quarter. Findings should be interpreted as representing the effect of the telehealth service model as delivered in routine practice rather than the effect of referral alone or any individual intervention component.

### Data

The study will use a cohort of monthly data from the Cheshire and Merseyside Secure Data Environment (CM-SDE), the NHS secure data environment that includes linked data across health and care services for all patients registered with a GP in Cheshire and Merseyside. We will use CM-SDE covering a period, 2018–2024 including linked data from the following datasets:

NHS

- Primary care data
- Primary care prescribing data
- Telehealth services data from the MC telehealth case management system.
- Secondary care data—Admitted Patient Care (SUS-APC)
- Community Services Data Set (CSDS)
- Mental Health Services Data Set (MHSDS)
- Emergency Care Data Set (ECDS)

Local Authority Adult Social Care

- Adult Social Care Dataset (ASCD)

Open data—linked to Lower layer super output areas (LSOAs)

- Indices of deprivation

All these datasets are available and can be linked within the CM-SDE. Patient characteristics will reflect individuals at the time of receiving telehealth service and so will correspond to the latest measurement taken during the study period 2018–2024. Table 2 summarises the details of outcome and exposure variables, definition criteria, and datasets from which the variable is derived that would be used in WP1 of the study.

**Table 2:** Exposure, covariates, and outcome variables for WP1.

Variable category	Variable	Definition/criteria	Data source
Primary exposure	MCTS enrolment	Active enrolment onto the Mersey Care Telehealth Service (Yes/No)	MCTS service dataset
Demographic and baseline characteristics	Age	Age at index date (≥18 years)	SUS-APC
	Sex	Male, Female, Other	SUS-APC
	Ethnicity	White British, Other White, Black, Indian, Pakistani, Bangladeshi, Chinese, Other Asian, Other ethnic group	SUS-APC
	Living alone	Yes/No	Primary Care
	Living with under-18s	Yes/No	Primary Care
	Household size	Number of people in household	Primary Care
	Geography	LSOA 2011	SUS-APC
	Deprivation	IMD 2019	SUS-APC
	Frailty	Fit, mild, moderate, severe frailty	Primary Care
Clinical characteristics	Long-term conditions	Hypertension, cancer, diabetes, CVD, heart failure, CKD, asthma, COPD, depression, schizophrenia, bipolar disorder	Primary Care
Baseline healthcare utilisation	Emergency admissions (previous 12 months)	Count of emergency admissions before index date	SUS-APC
Outcomes	Unplanned emergency hospital admissions	Count of admissions during follow-up	SUS-APC
	A&E attendances	Count of attendances during follow-up	SUS-ECDS
	30-day readmission	Readmission within 30 days of discharge	SUS-APC
	Mortality	Date of death during follow-up	Primary Care

**Abbreviations:** IMD—The index of multiple deprivation; A&E—Accident & Emergency; CVD—cardiovascular disease; CKD—chronic kidney disease; COPD—Chronic obstructive pulmonary disease; SUS-APC—Secondary uses service admitted patient care; SUS-ECDS—Secondary uses service emergency care dataset; LSOA—Lower layer super output areas

### Sample

Assuming effective 1:5 average weighting, this gives 19 million patient-months for analysis. Power analysis by simulation (Arnold et al., 2011), accounting for data and model structure indicates that this would be sufficient to estimate a medium effect size (Relative Risk = 0.85)—i.e., 15% relative reduction in the primary outcome attributable to the intervention, with 80% power across these subgroups.

### Analysis

The study uses two quasi-experimental methods: IPTW-DiD and RD designs. These methods help to reduce the risk of selection bias due to systematic differences between those receiving and not receiving the intervention, in two ways.

The primary approach of the analysis is IPTW-DiD, which is specifically designed to adjust observed differences by reweighting individuals according to their probability of receiving treatment, thereby approximating a pseudo-population in which treatment is independent of observed confounders. The treated cohort will comprise patients who received telehealth, with treatment timing defined by the first quarter in which telehealth was received; patient-quarter observations from this quarter onwards will be considered post-intervention. For untreated patients, comparable index quarters will be assigned to define the pre- and post-intervention periods.

The initial analytic cohort will be restricted to patients aged 50 years or above with at least one relevant long-term condition linked to the telehealth packages: COPD, diabetes, or HF. These eligibility criteria will be applied to both treated and untreated patients to ensure that the control group comprises individuals with a realistic probability of receiving the intervention. To avoid interpreting unobserved quarters as zero healthcare utilisation, the initial analysis will use a balanced panel of patients observed in all 24 quarters across the 6-year study period. The potential selection introduced by excluding patients who die, move away, or register with a GP partway through the study period will be explored in sensitivity analyses.

A treatment model will calculate the likelihood of each patient receiving the intervention using baseline socio-demographic variables, comorbidity measures, and 12-month cumulative pre-intervention healthcare utilisation, such as unplanned emergency admissions. For variables that may change over time, baseline or pre-intervention values will be used. Stabilised IPTW weights (Austin and Stuart, 2015) will be used in the DiD model to balance measured baseline covariates and reduce the influence of extreme weights. Covariate balance, weight stability, overlap/common support, effective sample size, and the plausibility of the conditional parallel-trends assumption will be assessed using weighting diagnostics and pre-intervention outcome trends. Comprehensive sensitivity analyses will be performed to evaluate the robustness of all estimates.

To manage the potentially large untreated population, the above eligibility restrictions will be applied before weighting. If the remaining comparison cohort is still too large for the available computing environment, a 5:1 propensity-score matched comparison cohort based on baseline characteristics may be constructed before applying the weighting approach; this will be reported as a computational sensitivity analysis.

Additionally, the RD design (Thistlethwaite and Campbell, 1960) will serve as a secondary complementary analysis, subject to the availability of a documented Johns Hopkins ACG risk score eligibility threshold (NHS England, 2025d) and sufficient observations close to that threshold. Threshold values, temporal changes, and geographical variations will be documented. As RD estimates a local effect among patients close to the eligibility threshold, its findings will be interpreted alongside, rather than as direct confirmation of, the IPTW-DiD estimates.

Furthermore, the subgroup analyses will be carried out to investigate heterogeneity in the impact of the telehealth intervention across socio-demographic groups. Depending on the size of the subgroup and the availability of data, these analyses will be conducted within the main IPTW-DiD framework using either stratified models or interaction terms. Age, sex, ethnicity, chronic health conditions, frailty score, IMD quintiles, and geographical locations are among the suggested subgroups. For each subgroup, propensity score models and weighting diagnostics will be re-evaluated to ensure appropriate balance. The RD subgroup analyses will be conducted only where there is sufficient sample size close to the eligibility threshold (NHS England, 2025d).

**Work package 2:**  *To determine whether patients using Mersey Care Telehealth Service report improvements in quality of health, mental health, satisfaction, and wellbeing, and whether this varies by sociodemographic group or service delivery type*

### Methods

Using a prospective study of 2,000 patients accessing MCTS and a comparison group of 2,000 who declined the service. We will gather survey data from telehealth patients on various health metrics, at 1-and 12-weeks post-enrolment, and we will contact patients, monthly, who have recently declined to use the service and collect baseline data and 12-week follow-up data for comparison.

The survey will include measures of health-related quality of life (EQ-5D-3L); Patient Health Questionnaire-9 (PHQ-9); Generalised Anxiety Disorder Questionnaire (GAD-7); Digital Health Literacy Scale (Nelson et al., 2022), and using MCTS standard survey questions, collect information on telehealth care support, patient satisfaction and evaluations on care quality and communication with healthcare professionals. Demographic information for MC telehealth patients is routinely collected. Thus, the survey for the comparator group will gather the following demographic information: health conditions (COPD, HF, and diabetes), age, sex, ethnicity, postcode, English language fluency, number of people living in the household, and reasons for declining the telehealth service for the comparison group to analyse deprivation indices. Our survey questions were based on those used by the Office for National Statistics (Office for National Statistics, 2023).

Our intervention group survey sample size is based on the size of the population of interest (people using the telehealth service), the level of confidence, and the degree of margin of (random) error. Our population is the approximately 22,000 patients who have used the MCTS. With a confidence level of 95%, a sample of 2,000 telehealth patients would provide a margin of error between 2% and 3% in how the responses would represent those of the total population. We feel that this is an acceptable margin of error. For a margin of error of 3% we would require 1,018 responses and so this allows for sufficient loss to follow-up from the target 2,000 participants. As such we require a similar size in the comparator group.

The inclusion criteria for the intervention group comprise patients who are receiving the MCTS and have a diagnosis of COPD, diabetes and/or HF. The comparison group consists of eligible patients who have declined telehealth services. Exclusion criteria for both groups include not having the aforementioned diagnoses, ineligibility for receiving MCTS, lack of mental capacity to consent, opting out of research, or not consenting to participate. Patients who decline telehealth may differ systematically from those who accept the service and will therefore not be considered a fully unbiased comparator group. Differences may include digital confidence, health literacy, illness perceptions, perceived burden of monitoring, social support, deprivation, language, disability, clinical severity, and previous experiences of healthcare. Baseline characteristics of accepters and decliners will be compared. Propensity weighting, matching or covariate adjustment methods will be used to reduce observable imbalances. Reasons for declining will be reported where available.

The comparator group will be recruited using the MC ‘Count Me In’ system (Bifarin et al., 2025; NHS Mersey Care, 2025). The ‘Count Me In’ system is an initiative to facilitate the involvement of patients in research. All patients can opt-out of the ‘Count Me In’ system. The research opportunity and participant information sheet will be provided in accordance with potential participants’ preferred method of contact.

For the intervention group, the survey forms part of routine practice and is administered through Docobo Ltd until March 2026; starting in April 2026, the Luscii system is used (NHS, 2025) thereafter (see MCTS section above). For the comparison group, the survey can be completed digitally (Jisc Online Surveys, i.e., https://app.onlinesurveys.jisc.ac.uk/login), by post or over the telephone. Where participants are completing the survey by telephone, researchers will input their answers on Jisc Online Surveys in real time on their behalf. Where participants are completing the survey by post, their answers will be recorded onto Jisc Online Surveys. Following data collection, data will be removed from Jisc Online Surveys and pseudo-anonymised for analysis.

### Analysis

Appropriate longitudinal analysis will be conducted using ANOVA and multilevel modelling (via R software or SPSS v26) to assess differences in self-reported outcomes between the telehealth intervention group and the comparator group. Key outcomes include adherence, digital health literacy, satisfaction with care, mental health indicators, and service use for primary and secondary care. Additionally, subgroup analysis will explore survey responses based on sociodemographic factors such as place, deprivation, ethnicity, age, and sex.

**Work package 3:**  *To interview patients and staff and understand the barriers and enablers of effective service delivery care*

### Methods

Remote (Microsoft Teams or telephone) semi-structured one-to-one interviews will be conducted with patients receiving the MCTS and staff delivering the MCTS or who are involved in the implementation of the MCTS. Informed by the Consolidated Implementation Framework for Research (Damschroder et al., 2009), two topic guides have been devised (one for patients, one for staff) in collaboration with Patient and Public Involvement (PPI) study team members (H.G., P.J.) and staff delivering the MCTS. Staff interviews will focus on participants’ professional roles, challenges, benefits, and suggestions for the telehealth system, as well as equity considerations and potential expansions to mental health services. Patient interviews will gather information about their health conditions, experiences with the MCTS, equity issues, and views on expanding the service to mental health care.

A total of 30 interviews, with *n* = 15 patients and *n* = 15 staff, will be held. Table 3 summarises the inclusion and exclusion criteria for each study population, Purposive sampling will be employed to increase the representativeness of our sample (Palinkas et al., 2015). We aim to interview patients with a range of different health conditions, patient-level socio-demographic characteristics (including deprivation and ethnicity), and experiences of accessing different monitoring levels (full, stepped down or light touch, and self-monitoring*)*. Moreover, we aim to interview staff with different roles and responsibilities who are involved in the implementation or delivery of the MCTS.

**Table 3:** The inclusion and exclusion criteria for each study population, and variables included in the sampling matrices.

Patients		Staffs	
Inclusion	Exclusion	Inclusion	Exclusion
MCTS patients diagnosed with HF, COPD and/or diabetes; included within the CMI system; consenting to participant	Not diagnosed with HF, COPD and/or diabetes; not listed on the CMI system; lack of mental capacity to consent as identified by MC staff, not consenting to participate	Staff who are involved in the implementation or delivery of the MCTS with or without patient contact, including nurses, healthcare assistants and administrative staff	Those who are not involved in the implementation or delivery of MCTS Those not consenting to participate.

**Abbreviations:** CMI—Count Me In; HF—Heart Failure; COPD—Chronic Obstructive Pulmonary Disease; MC—Mersey Care.

Patient participants will be recruited using Mersey Care’s ‘Count me in’ CMI system (NHS Mersey Care, 2025). The research opportunity and participant information sheet will be shared with potential patient participants according to their preferred method of contact. Staff participants will be recruited by email, through a study team member (P.A.), who is involved in the delivery of the MCTS. Informed consent (verbal or digital) will be obtained and recorded prior to interviewing. Interviews will be audio-recorded and transcribed verbatim.

### Analysis

Anonymised transcripts will be imported into NVivo-QSR 12 for analysis (Dhakal, 2022). Analysis will be informed by thematic approaches (Braun and Clarke, 2006) and the Consolidated Framework for Implementation Research (Damschroder et al., 2009). Survey responses from WP2 will be used to triangulate our qualitative analysis thereby increasing our understanding of patient experiences. Where participants have consented, member checking approaches will be employed to validate the main themes generated. PPI study team members (H.G., P.J.) will be involved in the analysis and advise on the interpretation of the findings.

**Work package 4:**  *To find out whether Mersey Care Telehealth Service is cost-effective*

### Methods

We will conduct a cost-utility analysis using quality of life data (RQ2), service costs, and information relating to healthcare resource use (RQ1). We will develop an economic model that considers health outcomes, health and social care service use (primary, secondary care, social care and care/nursing home admission), informal care costs and cost of resources, and estimates the incremental cost effectiveness ratio expressed as cost per quality adjusted life year in line with NICE guidance. The healthcare resource use will be measured from RQ1 and costs for the intervention and control group will be attached using NHS reference costs and Personal Social Services Research Unit reference costs for health and social care, possibly augmented with any local costs where necessary.

### Data and measures

Metrics for cost utility analysis will include quality of life measures (EQ-5D-3L from telehealth and WP2 survey data), service costs from Mersey Care, and health and social care use metrics. Additional measures may be taken as needed for model development. There is no applicable sample size or specific participant recruitment for this research question, as it relies on data collected for other research questions.

### Analysis

The model will be constructed using a 6 months-time horizon (within trial economic evaluation) and a 10-year time horizon using longer term follow up data for the programme and control groups. If there is not adequate person-years of follow up, then the data can be augmented with modelled estimates from other sources, such as English Longitudinal Study of Ageing and Health Survey for England. EQ-5D-3L score trajectories for control group will be estimated using Health Survey for England 2014 data and use Manca’s methods to control for differences in baseline utility (Manca et al., 2005). Health inequalities will be taken into consideration, and the model will account for outcomes disaggregated by IMD quintile. This may include carrying out distributional cost effectiveness analysis to look at cost effectiveness by IMD quintile.

## Patient and public involvement and engagement

Two public advisors (H.G., P.J.) are part of the research team contributed to the study design, reviewed study documents, and will be involved in the analysis, interpretation, and dissemination of our findings. H.G. & P.J. have lived experience of multiple long-term conditions, including mental health issues. They have extensive experience of being involved with research teams and working to embed a health equity approach to research via their work in NIHR Applied Research Collaboration North West Coast.

We will work together to complete the NIHR Health Inequalities Assessment Toolkit (rebranded into For Equity) (FOR-EQUITY, 2025) to ensure equity in telehealth access is maintained. It will be revisited regularly to ensure that issues of equity will be kept in focus throughout life of the project. To ensure that the project is inclusive and addresses the needs of people with mental health problems, we will work with the PPI team of the recently established Mental Health for Innovation (M-RIC) research facility (Mersey Care NHS Foundation Trust, 2025b).

Public advisors will be paid in accordance with the Involve guidelines. Training will be provided either via NIHR ARC NWC or on a one-to-one basis from research members of the team. PPI activities will be recorded and reported at the end of the study; the impact will be assessed using a bespoke, co-produced impact log and PPI reporting will be guided by the Guidance for Reporting Involvement of Patients and the Public (GRIPP2) tool (Staniszewska et al., 2017).

## Discussion

This protocol describes a multi-method study seeking to evaluate the effectiveness of the MCTS by assessing unplanned healthcare utilisation, patient-reported outcomes, implementation barriers and enablers, and cost-effectiveness. Telehealth has emerged as a key approach to support the transformation of the health system, especially in the NHS, where growing health disparities, multimorbidity, and service pressures have increased demand for digitally enabled care. To ensure that the implementation of telehealth is clinically effective, financially sustainable, equitable, and in line with the needs of patients and healthcare professionals, the study’s findings will support well-informed decision-making at the local, regional, and national levels (Department of Health & Social Care, 2022; NHS England, 2019a; NHS England, 2025b; NHS England, 2024a). This study examines health inequalities throughout. In WP1, we plan to explore deprivation, geographic location (place), ethnicity, age, sex, and service type as part of subgroup analyses. We will take a data-driven approach to define sub-groups to ensure that there is a sufficient number of participants within sub-categories to produce robust results. We will re-run the analyses within each sub-group to explore the impact of the telehealth service in the different groups. Within WP2, data permitting, we also plan to explore survey responses by deprivation, ethnicity, age, and sex. For WP3, we endeavour to employ a purposive sampling approach to interview a range of telehealth patients and staff. Furthermore, for WP4, we will consider outcomes across socioeconomic groups, stratified by IMD quintile.

### Limitations of the study

Several limitations are anticipated. First, the use of observational data introduces the potential for residual confounding, despite the use of propensity-score matching and multivariable modelling to mitigate these risks. Secondly, as this is a single-site study (Mersey Care NHS Foundation Trust), the findings may have limited generalisability. Third, routine clinical and administrative datasets may contain missing or incomplete data, particularly regarding socio-demographic variables or patient-reported outcomes. Qualitative components aim to mitigate this by including a diverse sample; however, some underrepresented voices may remain difficult to reach. The qualitative interviews may be influenced by selection bias, as participants who engage may have more positive experiences. Furthermore, the economic evaluation may be influenced by uncertainties in costing remote monitoring infrastructure, although sensitivity analyses will help to address this.

## Ethical and regulatory considerations

### Ethics approval

Ethical approval was obtained from the Health Research Authority and Health and Care Research Wales Ethics Committee (Ref: 25/NW/0268).

### Assessment and management of risk

A study risk assessment will be completed as part of the sponsor review process. It is important that people accessing the MCTS who are living with HF, COPD and/or diabetes can take part in our interview process, so we have a better understanding of their experiences of the service. However, although it is not anticipated that any interview questions will cause upset, it is possible that discussing their experiences might cause some distress, therefore we will ensure an ethically approved distress process is in place that has been co-produced with members of the Patient and Public Involvement and Engagement team.

### Peer review

To secure funding for this research programme, the application process involved two stages of scientific review conducted by the NIHR RfPB. In addition, we have an external peer review.

### Data protection/confidentiality

Cheshire and Merseyside have a Secure Data Environment that houses patient-level linked data for planning, population health, and research. Managed by Cheshire and Merseyside Integrated Care Board and hosted by the Data Service for Commissioners Regional Office through Arden & GEM Commissioning Support Unit, the SDE serves various stakeholders, including commissioners, local authorities, providers, and researchers. Built on Azure Cloud, the SDE provisions data on a project-by-project basis using an ‘Airlock’ system, ensuring all analysis occurs within the environment, and data cannot leave without approval under strict minimisation criteria.

The SDE integrates data from three main sources: National Commissioning Datasets from NHS England, local provider data flows, and the Cheshire and Merseyside Shared Care Record managed by Graphnet. All data within the environment is de-identified, removing direct identifiers while maintaining pseudonymised records for research use. Data access is granted on a project basis through the Cheshire and Merseyside Data Access and Asset Group, which includes representatives from data controllers, processors, a Data Protection Officer, Caldicott Guardian, and public members. Applicants must submit a Data Access Request Form detailing project scope, legal basis, datasets, data flow, and governance measures. Individuals retain the right to opt out of data sharing through nationally defined processes.

Participant confidentiality will be maintained in accordance with the Data Protection Act 2018, UK General Data Protection Regulation, and applicable data protection legislation. Mersey Care NHS Trust is the data controller. Researchers at the University of Liverpool will collect participant names and informed consent records for the qualitative studies involving MC telehealth staff (including nurses, healthcare assistants, and administrative staff), patients receiving the telehealth service, and patients who declined the service. All data will be managed in accordance with NHS Research Ethics Committee–approved procedures.

## Dissemination policy

This study aims to evaluate the clinical effectiveness, cost-effectiveness, and acceptability of the MCTS, while exploring opportunities for future service development, including potential expansion into mental health. Evidence from patients, the public, and healthcare staff will help inform telehealth implementation and service planning both locally and nationally. Findings will be disseminated through open-access publications, easy-read summaries developed with support from the PPI group, patient and public networks, newsletters, websites, and stakeholder workshops. Results will also be shared with key organisations and professional bodies, including NHS England, the Mersey Care Executive, the Royal College of General Practitioners, and the Royal College of Physicians. In addition, findings will be presented at academic conferences and published in consumer-focused outlets such as Heart Matters (British Heart Foundation) and Positive Pressure (Blood Pressure UK) to reach current and prospective telehealth users.

## Supplementary Material

Supplementary_materials_oqag032

## Data Availability

The study datasets are not publicly available due to data protection and governance restrictions. This study will use the data through the Cheshire and Merseyside Secure Data Environment (CM-SDE), an NHS Secure Data Environment that provides linked, de-identified health and care data for all patients registered with a GP in Cheshire and Merseyside. Access to CM-SDE data is permitted only within the secure research environment and requires approval through the appropriate NHS data access and governance processes. Researchers wishing to access these data must submit a request via the CM-SDE governance team and obtain approval through the required information governance, ethical, and/or Data Access Request procedures. All analyses will be conducted within the secure environment, and only aggregate, non-disclosive outputs were permitted to leave the CM-SDE. The datasets generated and/or analysed for the qualitative study are maintained in our institutional repository. Subject to participant consent and ethical approval, de-identified data may be made available upon reasonable request to the corresponding author.
