# Supplementary material for: Telehealth to reduce compound pressures on the National Health Service and social care: a multi-methods study protocol
Source: Oxf Open Digit Health. 2026 Sep 18;4:oqag032. doi: 10.1093/oodh/oqag032 (PMC13592275; doi:10.1093/oodh/oqag032)
Supplement: Supplementary file 1 [file supplementary_materials_oqag032.pdf]

GLOSSARY OF TERMS RELATING TO TELEHEALTH

Term	Definition
Case finder	The member of VW (Virtual Ward) Team tasked with identifying those patients suitable for admission to a VW.
Clinical lead	The lead Dr/Consultant for the specific VW
COPD	Chronic Obstructive Airways Disease - a respiratory disorder resulting in severe/disabling breathing problems
DAAG	Data Asset and Access Group describes a system to limit or grant access to specific data records for data security purposes
Difference in difference methods	Compares the average change in an outcome over time in an intervention group to the average change over time for a control group.
Digital Buddies	Digital Buddies were volunteers who helped, during the COVID19 pandemic, to enable older people to connect digitally with friends, family, groups,& the wider world. May have a role in Telehealth
Docobo	Is the current provider of the technology used to send patients results to the telehealth hub and the system they use to monitor and track all patients and staff.
DM	Diabetes Mellitus - disorder of sugar metabolism, Type I - the body doesn't produce enough insulin from the pancreas. Type II - The body doesn't produce enough insulin for the needs of the cells, or the body's cells don't respond properly to the level of insulin produced.
EQ-5D-3L	EuroQol five-dimensional questionnaire is a standardised instrument for measuring health related quality of life
ForEquity: Focus on Research and Equity	A suite of tools and resources developed by ARC NWCOAST to help make research evidence more relevant for action to reduce social and health inequalities.<br><br>FOR-EQUITY – tools and resources to help reduce social and health inequalities (forequity.uk)
Health inequalities assessment tool kit	Part of the ForEquity tool kit. It is a prompt tool for research teams to reflect on the equity dimensions of a topic/project and how these can be integrated into the design, conduct and reporting of the research.
HF	Heart Failure clinical term for a condition where the heart is unable to pump blood around the body properly and needs treatment to help it work.
Hospital at home (HaH)	Admission-avoidance Hospital at Home care, provides co-ordinated, multi-disciplinary, and integrated care in the home for people who would otherwise be admitted to hospital. A blend of in person care and Virtual Ward<br>Home (hospitalathome.org.uk)
ICB	A statutory NHS organisation responsible for developing a plan for meeting the health needs of the population, managing the NHS budget and arranging for the provision of health services in the ICS area.<br><br>NHS England » What are integrated care systems?
ICS	Integrated care systems (ICSs) are partnerships of organisations that come together to plan and deliver joined up health and care services, and to improve the lives of people who live and work in their area.<br><br>NHS England » What are integrated care systems?
Long Term Condition (LTC) monitoring at home	Differs from VW in that can be used to support/manage chronic (long term) health care needs
LoS<br>ALoS<br>RLoS	Commonly used acronyms referring to stay in hospital or VW<br><br>Length of stay<br>Average length of stay<br>Reduced length of stay

Term	Definition
MCFT	Mersey Care Foundation Trust
MLTC	Multiple Long Term Conditions, also referred to as multi-morbidity
Multi-morbidity	Multiple Long Term Conditions, term used by clinicians but disliked by patients and public
On-boarding	Describes getting a patient onto the Telehealth system
Place based partnerships	Within each ICS, place-based partnerships will lead the detailed design and delivery of integrated services across their localities and neighbourhoods.<br><br>NHS England » What are integrated care systems?
PHQ-9 and GAD-7	Both Measures of depression and anxiety
QALY	Quality Adjusted Life years - a calculation used in Health Economics
Site lead	The lead person charged with overseeing the VW in your area
SOPs	Standard Operational Protocols
Technology Enabled Care (TEC)	Technology Enabled Care (TEC) includes Telehealth, telecare, telemedicine, tele-coaching and self-care services that put people in control of their own health, wellbeing and support, keeping them safe, well and independent and offering them and their families peace of mind.<br><br>TSA - the voice of TEC (tsa-voice.org.uk)
Telehealth	Broad term used to describe a range of telecommunication technologies and strategies to provide healthcare from a distance rather than face to face.
Telehealth levels of Monitoring	**Full Monitoring** - Daily monitoring of a range of parameters -dependent upon the condition affecting the patient - eg Heart Failure Usually 5-6 months<br>**Step-down** - less intensive monitoring, often less in frequency, may include fewer parameters and may continue for a further 6 months or longer<br>**Light-touch monitoring** May be weekly or less frequent often up to 12 months can follow on as part of step down or be a stand alone option.<br>**Self-directed monitoring** - may be ongoing monitoring by the patient keeping a record of a number of parameters that may be self determined.<br>Patients may move up and down these levels of monitoring determined by clinical need.
Telehub	Site where the healthcare practitioners deliver care through telecommunication systems. Results are sent in by patients and practitioners, they are monitored and inform decision making and subsequent treatment. This is usually the Point of contact for patients and their families/carers.
Telehealth hub staff	The staff who man the phones and monitor the results sent in by patients and clinicians. Multi-disciplinary team (meaning clinical and non-clinical staff from a range of specialties and backgrounds)
Virtual Ward -VW	Virtual wards allow patients to get the care they need at home safely and conveniently, rather than being in hospital.
